# Dephasing-Induced Control of Interference Nature in Three-Level Electromagnetically Induced Tansparency Systems

**DOI:** 10.1038/srep16370

**Published:** 2015-11-16

**Authors:** Yong Sun, Yaping Yang, Hong Chen, Shiyao Zhu

**Affiliations:** 1 Key Laboratory of Advanced Micro-structure Materials (MOE) and School of Physics Sciences and Engineering, Tongji University, Shanghai 200092, China; 2 Beijing Computational Science Research Center, Beijing 100084, China; 3 Synergetic Innovation Center of Quantum Information and Quantum Physics, University of Science and Technology of China, Hefei, Anhui 230026, China

## Abstract

The influence of the dephasing on interference is investigated theoretically and experimentally in three-level electromagnetically induced transparency systems. The nature of the interference, constructive, no interference or destructive, can be controlled by adjusting the dephasing rates. This new phenomenon is experimentally observed in meta-atoms. The physics behind the dephasing-induced control of interference nature is the competing between stimulated emission and spontaneous emission. The random phase fluctuation due to the dephasing will result in the correlation and anti-correlation between the two dressed states, which will enhance and reduce the stimulated emission, respectively.

Coherent processes based on the quantum interference in three-level media have been investigated for many years^1^,^2^,^3^,^4^,^5^,^6^,^7^,^8^,^9^,^10^,^11^, and yielded many novel and unexpected phenomena, for example, electromagnetically induced transparency (EIT)^3^,^4^,^5^, lasing without inversion (LWI)^6^,^7^,^8^, and spontaneous emission cancellation^9^,^10^. Alkali metals in gas phase were employed in the first and many recent experiments^3^,^11^, for their simple electronic level structure and long-lived coherence. Later experiments were also conducted with solid system, such as doped solid^12^, quantum dots^13^, and superconducting circuits^14^. The EIT brings the absorption cancellation (or reduction) at the resonant frequency of a transition, and gives rise to steep dispersion, as well as greatly enhanced nonlinear susceptibility in the spectral region of induced transparency of the medium^5^. Now, EIT has become a crucial technique for its potential applications such as slowing of light^15^, optical storage^16^, quantum information processing^17^, and optical diodes^18^.

Two methods, bare states and dressed states^3^,^5^, are used in analyzing the EIT in closed three-level atoms, where a weak field probes a transition between two level and a strong field couples the upper level of the transition to the third level. In the bare state method, the absorption reduction is due to multiple pathways through the strong field coupled transition many times^19^. In the dressed state method, the EIT results from a combination of Autler-Townes splitting of the two dressed states and destructive interference in the probe absorption due to the dressed states^20^. Most of the studies on the upper-level coupled three-level atoms claim that there is only destructive interference, while for the lower-level coupled three-level atoms, most claim no interference^5^,^21^. Comparisons between the EIT and Autler-Townes splitting show that the quantum interference is important when the Rabi frequency of the coupling field is at the order of decay rates^21^,^22^,^23^. When the Rabi frequency is much larger than the decay rates, the two peaks of the absorption spectrum are well separated, and the difference between the EIT and the Autler-Townes splitting (without interference) is very small, especially for the resonance absorption. However, the influence of the dephasing on the interference nature has not been discussed. In Ref. 24, the Author gave an approximate equation under the condition of the Rabi frequency of the coupling field much larger than the decay rates, from which he found constructive (destructive) interference for lower-level (upper-level) coupled three-level atom, if the dephasing is neglected. Although, he did not explicitly discussed the transfer of the interference nature (between constructive and destructive), this equation indicates that the transfer is possible by using the dephasing rates. Furthermore, we would like to reveal the physics behind the control of interference nature.

The classical analogies of the EIT based on artificial meta-atoms recently have attracted a lot of attention^25^,^26^,^27^,^28^,^29^,^30^,^31^,^32^,^33^ due to easy experimental demonstration and potential applications, such as slow light^25^,^26^, low-loss metamaterial^27^,^28^, optical storage^30^, and sensing^31^. For the meta-atoms, not only the coupling strength (corresponding to the strength of the driving field), but also the intrinsic parameters of the systems can be adjusted in a relative easy way, such as the dephasing rates by using variable resistors^34^,^35^. Therefore, meta-atoms provide a flexible experimental platform to simulate the quantum interference phenomena in multi-level atomic systems.

In this work, we discuss the systematical control of the quantum interference nature by changing the dephasing rates in the EIT three-level system (the upper level coupled by a strong field) with the Rabi frequency of the coupling field at the order of the decay rates. Our dephasing-induced control of interference is established with the two dressed states. The dynamic equations for the two dressed states clearly show that the correlation between the two dressed states, and consequently the nature of the quantum interference, is determined by the decay rates, so that one can have constructive, destructive or no interference in the atomic absorption spectrum by simply changing the decay rates, particularly the dephasing rates. Here we also report the first experimental observation of the dephasing-induced control of the interference nature in the EIT three-level meta-atoms. The dephasing-induced transfer from constructive interference to destructive interference is demonstrated. Our results provide a clear understanding of the nature of quantum interference in three-level EIT systems, and pave a new way to control all kinds of interference by adjusting the dephasing rates.

## Results

### Dephasing-induced control of interference nature

**Figure 1:**
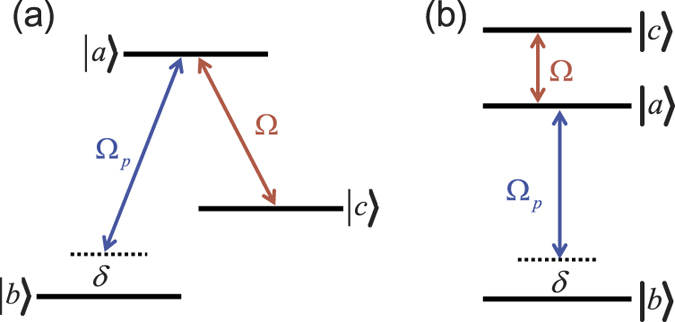
Three-level schemes with resonant driving. (**a**) EIT-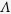 type. (**b)** EIT-cascade type. 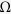 and 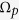 are the Rabi frequencies of the coupling and probing fields, respectively. 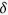 denotes the frequency detuning.

Consider the upper-level coupled three-level systems, which can be EIT-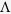 [see Fig. 1(a)] or EIT-cascade [see Fig. 1(b)] schemes. The three-level atom is driven by a strong field with frequency 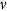 and Rabi frequency Ω, and is probed by a weak field with frequency 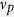 and Rabi frequency Ω_*p*_. The Hamiltonian is 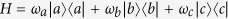

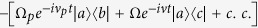
. Setting 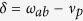 and 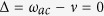 (resonant driving), the Hamiltonian in rotating frame can be written as 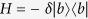

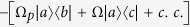
 for the 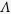 scheme. The Hamiltonian for the cascade is the same with 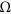 replaced by 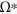. The density matrix equations for 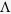 scheme in bare states are


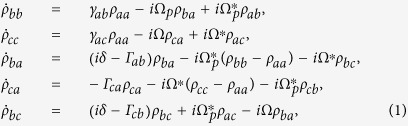


where 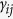 is the population decay rate from 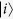 to 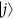, and 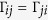 is the off-diagonal decay rate of 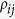. Note 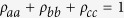. The equations for the cascade scheme are the same with 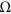 replaced by 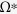, and the second equation replaced by 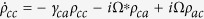.

The imaginary part of the atomic linear susceptibility, or the absorption, is proportional to the real part of 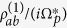, which can be obtained by perturbation method and is the same for both schemes,


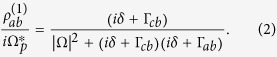


Let us consider the dressed states 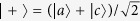 and 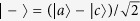. The density matrix equations in the dressed states for both schemes can be obtained from Eqs. (1),


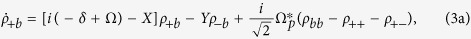



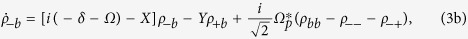


where 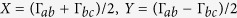. With the zeroth order solution 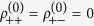 and 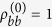, we can obtain


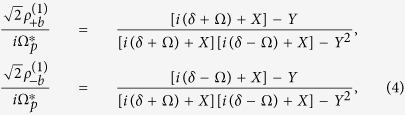


and the absorption becomes 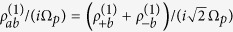.

If 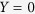, Eqs. (3a) and (3b) are two independent equations, and have no correlation between them, and Eqs. (4) tell us that the emission spectrum is a sum of two independent Lorentzian peaks with the same linewidth 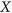 and there is no interference. *Y* is responsible for the correlation between 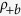 and 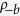, and the interference in the absorption. The energy difference between the two dressed states is 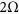 determined by the driving field. We can consider that the parameter 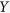, which can be written as 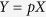 (−1 < *p* < 1), describes the correlation between the two effective dipole moments (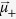 and 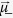) of the two transitions. Such dipole correlation represented by 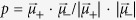 will result in the quantum interference phenomenon^9^. For 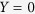 (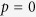), we have the two dipole moments orthogonal and no interference. For 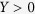 (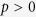), we have an acute angle between the two dipole moments, and the absorption around 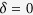 is less than the sum of the two Lorentzians, which means the destructive quantum interference in the absorption. For 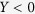 (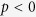), we have an obtuse angle between two dipole moments, and the absorption around 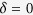 is more than the sum of the two Lorentzians, which means the constructive interference in the absorption. As 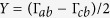, we have 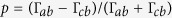. We can see that 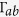 and 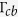 have different roles in the interference: destructive (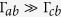 and 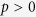) and constructive (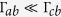 and 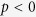), respectively.

**Figure 2:**
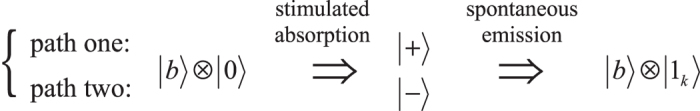
The interference pathways in EIT system. The two pathways, which form the interference pair, are stimulated absorption (from 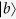 to 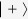 and 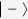), and the spontaneous emission (from 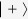 and 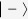 to 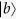).

As *Y*, which depends on the decay rates, is responsible for the interference in the absorption, it is clear that only the stimulated absorption from the lower level 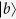 to 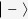 and from 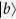 to 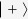 (from one state to two different states) could not form the pair of the interference pathways. The two interference pathways must include spontaneous decay process. Therefore, the two pathways, which form the interference pair, are stimulated absorption (from 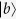 to 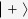 and 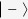), and then spontaneous emission (from 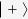 and 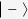 to 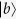), see Fig. 2. It is the interference of the spontaneous quenching together with the stimulated absorption that makes the electromagnetically induced transparency.

**Figure 3:**
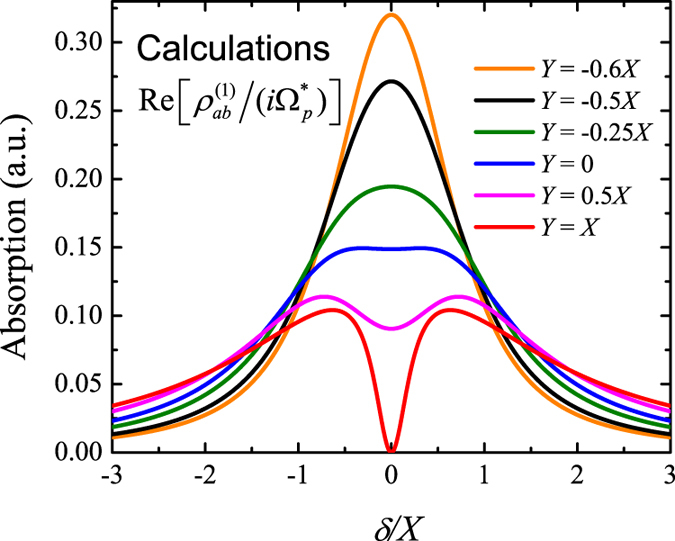
Calculated absorption spectra for upper-level coupled three-level system, which is proportional to the real part of 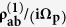. The magnitude of absorption at the resonance frequency gradually decreases with 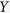. For 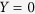, the absorption spectrum equals to the sum of two Lorentzian peaks with the same linewidth 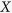 (Blue). For 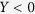 (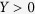), constructive (destructive) interference of the absorption is observed at the resonance frequency.

For the 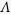 scheme we have 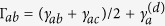 and 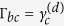, with 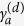 and 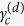 the dephasing rates for 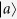 and 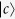, respectively. If the dephasing rate 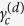 is very small compared with 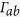, we have 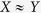, leading to almost complete destructive interference at 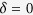. If we have very large dephasing rate 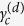 compared with 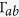, which can be realized by adding some collision mechanism with level 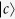 (not level 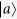), we will have 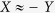, resulting in almost complete constructive interference at 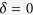. By changing the dephasing rate 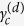 from small to large, we can have from almost complete destructive interference to no interference, and then to constructive interference. In Fig. 3, we plot the real part of 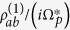 for the 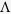 scheme with different 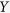 from 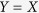 to 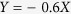 with 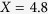 and 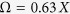. The blue curve is a sum of two Lorentzians for 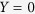. From Fig. 3 we can see clearly the transition from complete destructive interference (red curve) to constructive interference (orange curve). Similar curves can be found for the cascade EIT scheme, from almost complete constructive (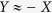) to almost complete destructive (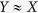) interference by changing only the dephasing rates.

We ask ourselves why 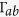 and 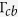 play the different roles in the interference and what is the mechanism to understand the dephasing-induced control of interference. The decay rates 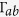 and 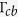 are related to the population decay and dephasing of level 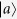 and 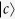, respectively. The decay 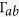 is caused by population and dephasing reservoirs of 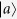, while decay 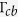 is caused by population and dephasing reservoirs of 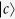. The reservoirs of 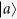 (or 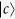) result in random phase changes of 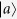 (or 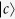). The random phase changes of 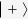 and 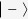 due to the reservoirs of 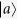 are correlated by the strong field, so that the two random phase changes are of the same magnitude and the same sign because of  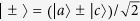 (the common plus sign before 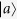). Due to the correlation of the random phase changes for 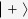 and 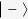, the system from 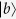 to 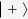 and 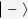 by stimulated absorbing a probe photon can stimulatedly emit the same probe photon with the same frequency and phase from to 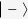 and 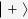 to 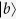, which has no contribution to the absorption. It is these stimulated emission processes that prevent the spontaneous emission, so that the contribution of the reservoirs of 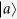 to the spontaneous emission is reduced or eliminated. This is why we have destructive interference. Similar discussion can be made for the reservoirs of 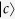 , which will result in anti-correlation (same magnitude with opposite signs) for the random phase changes, because of  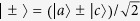 (the opposite signs before 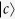), which leads to reduction or inhibition of the stimulated emission and enhances the spontaneous emission, so that we have constructive interference in the absorption. For 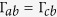 (Y = 0), the correlation and the anti-correlation cancel each other, and we have no interference.

### Experimental demonstration in meta-atoms

For an atomic system, the adjustment of the dephasing rates is limited because it is difficult to get proper parameters by direct tuning the atomic collisions^36^,^37^,^38^. Here we resort to the classical analog of EIT in meta-atoms to give the experimental demonstration of the dephasing-induced control of interference nature.

It has been shown that the dynamic equations for the classical EIT in meta-atoms are given as^26^,^33^,^39^,^40^


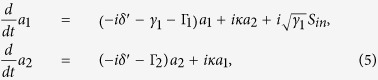


where 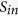 is the input field with frequency detuning 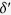 which will excite the mode 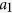 (but not the mode 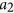), and 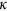 is the near-field coupling strength between the two resonant modes in the meta-atom (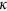
’s role is same as the Rabi frequency 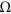 in atomic EIT system). 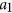 is a “bright” mode that has outputs in both forward and backward directions with a scattering loss rate 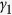, while 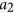 is a “dark” mode that has no output and no scattering loss. The two modes have the same resonant frequency, and suffer decay with different dephasing rates 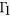 and 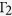, respectively.

After some derivations, we obtained that


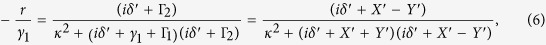


where 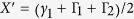, 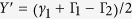, 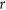 is the reflection coefficient, and the transmission coefficient is 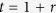. The dependence of 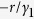 on the decay rates of the resonant modes (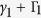 and 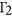) is the same as that in 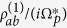 on the atomic decay rates, see Eqs. (2) and (4). Therefore, the real part of 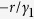 corresponds to the absorption in the atomic EIT system. We can measure *r* versus 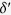 to simulate the dependence of the absorption in the atomic EIT system on 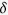.

**Figure 4:**
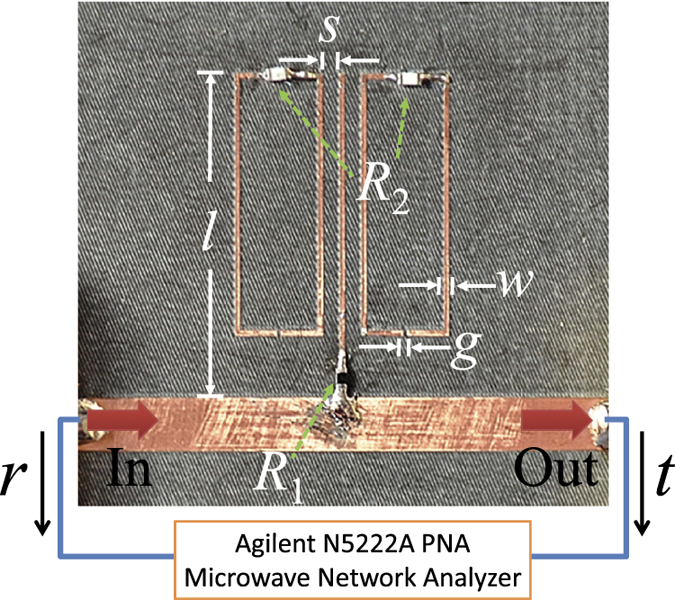
Experimental setup to simulate the dephasing-induced control of interference in atomic EIT system. Transmission and reflection coefficients are measured with the microwave network analyzer. The EIT meta-atom consists of a “bright” resonator, a “dark” resonator, the pair of split rings. The near-field coupling strength between the two resonators is determined by the separation 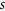, and the dephasing rates of the two resonators, 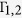, can be adjusted by two resistors 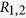, respectively. Geometric parameters can be found in the Main Text.

Our experiments are conducted by using meta-atom in microwave range. The experimental setup is shown in Fig. 4. The meta-atom is composed of two coupled resonators. The bright resonator (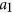) is a copper branch with the length of 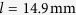, and it is connected with the main-strip by the resistor 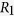, as indicted in the middle of Fig. 4. The dark resonator (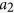) is composed of two metal split rings with the dimensions of 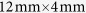, which are located at the two sides of the first resonator. It has been demonstrated that the response of this configuration composed of the bright and the dark resonators can be regarded as the classical analogue of the EIT^26^,^27^,^28^,^29^,^34^,^35^. Here the width of the metal copper wires is 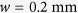. The gap size of the two rings is 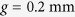. The frequencies of the two resonant modes are designed to be the same at 23.56 GHz. The dephasing rates of the two resonators, 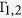, can be adjusted by two resistors 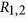, respectively. By putting the two resonators close to each other with a distance of 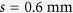, we can introduce the near-field coupling 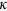 between the two modes. When a microwave of frequency 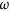 propagates along the main-strip (incident from the left), we have input to the bright resonator (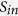) which excites 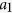. The dark resonator is far away from the main-strip, so that no input for the dark resonator. The dark mode 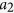 is excited by the coupling between the two modes. The motion of the resonant modes is described by Eq. (5). After doing the full-wave simulations of the meta-atom with the chosen geometric parameters, we have 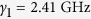, 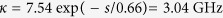, and 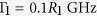, 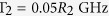
39. Then we can adjust 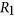 and 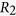 to have different 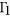 and 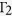.

**Figure 5:**
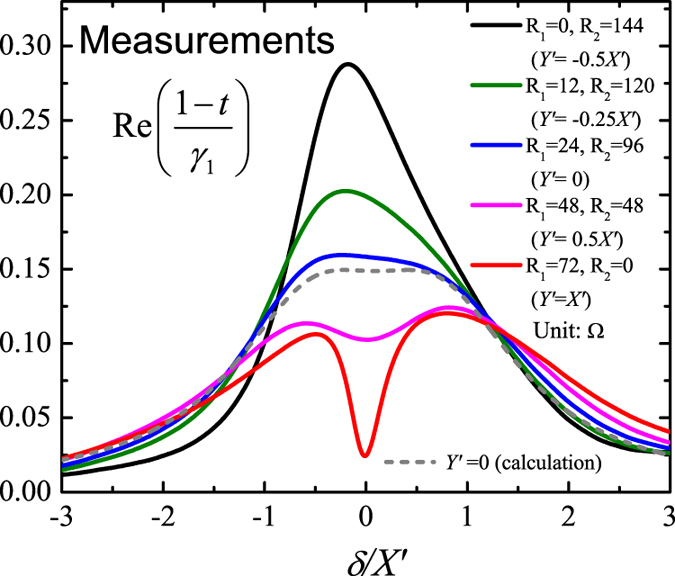
Measured spectral of the real part of 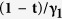 with different dephasing rates given by resistors R_1,2_. The real part of 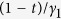 corresponds to the the real part of 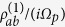. As a comparison, the theoretical calculation for 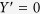 is given with the gray dash line. Our measurements consist with the calculations in Fig. 3 reasonably well.

In Fig. 5, we present the measured spectral of the real part of 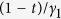 (rather than the equivalent quantity 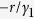) with different dephasing rates given by resistors 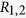. This is mainly for two reasons: (i) Compared to the reflection coefficient *r* with respect of the mirror plane of the structure, the transmission coefficient *t* is relatively easy to measure. (ii) The slight asymmetry of the sample due to the fabrication leads to the difference between the reflections for the incident from left side and right side, while the transmissions are reciprocal and uniquely determined. Here we fix 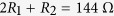, which makes 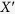 be a constant, 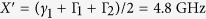, and all of the frequencies and dephasing rates are normalized with 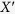. By changing *R*_1_ and *R*_2_ simultaneously, we can make 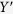 from 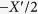 (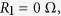

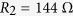
) to 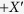
(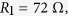

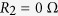
), that is to say, from a constructive interference to a destructive interference. The measured spectra of 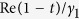 in Fig. 5 show clearly the phenomenon of the dephasing-induced control of interference, namely, the transition from the constructive to the destructive interference with the increasing of 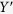. For the case of 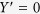 (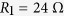 and 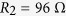), there is no interference, and the measured spectrum approaches to the sum of the two Lorenzians, see the blue curve (the theoretical calculation is also plotted for comparison, see the gray dashed curve), with the resonance centers 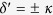 and the common line-width 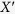. Here non-zero value of 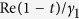 at 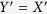 (red curve in Fig. 5) is due to a little inevitable dissipation from the material and the roughness. Although there is a little difference between our measurements and theoretical calculation due to the fabrication tolerance, as well as the effects from the high-order modes and the imperfect match at input/output ports (as these unfavorable factors are not considered in theoretical calculation), our experiments have no doubt simulated the constructive, destructive and no interference in EIT three-level system by adjusting only the dephasing rates in meta-atom.

## Discussion

The new phenomenon, the dephasing-induced control of interference nature has been theoretical investigated and experimentally observed in the three-level EIT system. We find that the nature of the interference is dependent on the decay rates, but not related to the strength of the driving field. The random phase fluctuation due to the dephasing will result in the correlation and anti-correlation between the two dressed states, which will enhance and reduce the stimulated emission, respectively. The physics behind the dephasing-induced control of interference is the competing between stimulated emission and spontaneous emission.

## Methods

The samples are all fabricated on copper-clad 0.787-mm thick Rogers RT5880 substrates using laser direct structuring technology (LPKF ProtoLaser 200). Transmission and reflection properties were obtained directly from the microwave vector network analyzer (Agilent N5222A). In addition, a commercial software package (CST Microwave Studio) is used in designing the samples.

## Additional Information

**How to cite this article**: Sun, Y. *et al.* Dephasing-Induced Control of Interference Nature in Three-Level Electromagnetically Induced Tansparency Systems. *Sci. Rep.* **5**, 16370; doi: 10.1038/srep16370 (2015).
